# The Impact of Cognitive Testing on the Welfare of Group Housed Primates

**DOI:** 10.1371/journal.pone.0078308

**Published:** 2013-11-06

**Authors:** Jamie Whitehouse, Jérôme Micheletta, Lauren E. Powell, Celia Bordier, Bridget M. Waller

**Affiliations:** 1 Department of Psychology, Centre for Comparative and Evolutionary Psychology, University of Portsmouth, United Kingdom; 2 Marwell Wildlife, Winchester, United Kingdom; Université Pierre et Marie Curie, France

## Abstract

Providing cognitive challenges to zoo-housed animals may provide enriching effects and subsequently enhance their welfare. Primates may benefit most from such challenges as they often face complex problems in their natural environment and can be observed to seek problem solving opportunities in captivity. However, the extent to which welfare benefits can be achieved through programmes developed primarily for cognitive research is unknown. We tested the impact of voluntary participation cognitive testing on the welfare of a socially housed group of crested macaques (*Macaca nigra*) at the Macaque Study Centre (Marwell Zoo). First, we compared the rate of self-directed and social behaviours on testing and non-testing days, and between conditions within testing days. Minimal differences in behaviour were found when comparing testing and non-testing days, suggesting that there was no negative impact on welfare as a result of cognitive testing. Lipsmacking behaviours were found to increase and aggressive interaction was found to decrease in the group as a result of testing. Second, social network analysis was used to assess the effect of testing on associations and interactions between individuals. The social networks showed that testing subjects increased their association with others during testing days. One interpretation of this finding could be that providing socially housed primates with an opportunity for individuals to separate from the group for short periods could help mimic natural patterns of sub-group formation and reunion in captivity. The findings suggest, therefore, that the welfare of captive primates can be improved through the use of cognitive testing in zoo environments.

## Introduction

Introducing cognitive challenges into captive environments is increasingly recognised as a potential method to enhance the welfare of captive animals through the enriching effects of problem solving [1]. These benefits have been documented when presenting animals with both naturalistic problem solving opportunities, such as elaborate foraging mechanisms, and more recently, non-naturalistic problem solving opportunities through the interaction with computerised devices [2]. The enriching effect of such devices has been particularly documented in the primates [3], [4] with some species being observed to seek interaction with mentally stimulating equipment without food reward or other extrinsic positive reinforcement [5]. Most of the studies to date assess the welfare impact of devices which were developed with the specific aim to increase enrichment. Increasingly, however, cognitive tasks are being presented to captive zoo animals primarily for cognitive research, where the welfare benefits are important but secondary to the main scientific aims. It is important to assess, therefore, whether those same welfare benefits can be achieved through the use of devices developed for the purpose of cognitive research. Empirical studies examining the impact that cognitive testing can have on the behaviours of group housed primates are essential before we can claim that they can result in positive effects on welfare.

Measuring welfare quantitatively through the observation of self-directed and social behaviours is a useful and standard methodology in welfare studies [6]. However, individuals respond to changes in the environment differently, particularly when they are part of a complex social group governed by dominance hierarchies, kinship and friendship alliances. Therefore, additional measures examining the members of a social group independently may be beneficial when addressing questions about welfare [3]. Social Network Analysis (SNA) could be a useful tool to overcome this problem [7]. SNA allows complicated group level and individual changes in social dynamics to be characterised and visualised over both long periods of time [8] and between short-term environmental or social changes. Through comparisons of associations and behavioural interaction, we can use SNA to look at group structure beyond dyadic interactions alone, and instead look at the topology of the entire group [9]. Assessing the impact on individuals in this way may be particularly important when cognitive challenges are presented as part of cognitive experiments (rather than enrichment devices per se) as individuals separate from the group. Thus, how individuals (and the rest of the group) respond to this process may differ.

Examining the welfare impact of cognitive testing on captive animals is important as this method is increasingly proposed as an alternative to laboratory based testing. In addition to welfare benefits, there may also be scientific advantages. Many cognitive mechanisms in non-human primates are unable to be described by simple observation and need to be assessed through more controlled experimentation [10] and research laboratories are currently the favoured setting for these ex-situ cognitive studies. However, despite the benefits of highly controlled test conditions, laboratories can potentially lead to inhibition in the development of natural cognitive functions which would normally be seen as a product of complex social and environmental conditions [11]. Cognitive testing of subjects raised in enriched environments which try to simulate naturalistic conditions such as zoo’s and wildlife parks therefore could provide advantages to laboratories and allow us to make more appropriate assumptions about the cognitive abilities of animals in natural populations [12]. Some inhibition of cognitive development in captive environments cannot be ruled out completely, as these environments are rarely able to replicate the ecological or social conditions as observed in the wild.

The use of single or pair-housed laboratory subjects also has limited value when addressing questions regarding the impact of social factors on cognition due to partial or complete social deprivation. Socially housed primates as subjects of experimental testing can therefore provide unique opportunities to ask research questions about social influences on cognition. Indeed, many aspects of behaviour, such as dominance, kinship and friendship, are already understood to have significant effects on cognition [13], [14].

Importantly, Gazes *et al.* [11] demonstrated that the uncontrollable environmental factors which are inevitable when testing with group housed primates did not cause scientific results to be affected adversely. Both pair and group housed rhesus macaques (*Macaca mulatta*) performed comparably when given identical tasks, an effect also reported in other studies [15]. Primates housed in more natural social groups and environmental conditions are also in much higher abundance throughout zoos and wildlife parks than in laboratories, with a much greater choice of primate species. This provides better opportunities to study cognition from a comparative perspective, and also allows the study of endangered and lesser known species [14]. The use of these rare species in cognitive science could facilitate the study of other aspects of their biology, which in turn could have implications for their conservation.

Here, we assessed the impact of cognitive testing on the welfare of zoo-housed macaques (the Macaque Study Centre, Marwell Zoo, Hampshire, UK) through comparison of behavioural rates and analysis of the social network in testing and non-testing conditions.

## Methods

### Ethics Statement

This study was carried out after receiving approval by the University of Portsmouth regulated by the Department of Psychology Animal Ethics Committee, and Marwell Wildlife’s Ethics Committee. Subjects were never food deprived for the cognitive tasks conducted alongside this study, always entered the testing room voluntarily and were kept to normal daily husbandry schedules predetermined by zoo staff throughout the observation period.

### Subjects and Housing

This study was conducted between June 2012 and January 2013. Subjects were a social group of crested macaques (*Macaca nigra*, n = 5, 4 females) ranging in ages from 8–30 years old. Three were actively involved in non-invasive experimental research at the Macaque Study Centre, at Marwell Zoo, Hampshire, UK. This research facility is an extension to the macaques’ normal enclosure and allows the voluntary participation of individuals in cognitive tests when a researcher is present. The subjects involved in testing were trained to participate in matching-to-sample tasks using a touch-screen [16]. Cognitive testing required subjects to break from their social group and enter a specially built testing area. When referring to cognitive testing in this paper, we are including all aspects of the procedure - both the voluntary splitting of the individual and the participation in tasks. Subjects had access to an indoor (5×5 m, 4 m high), outdoor (10×5 m, 4 m high), an isolated and sheltered testing area (1×2 m, 4 m high) and an island (15×15 m). The testing area was located within the outdoor enclosure at the opposite end to their indoor sleeping areas. All parts of the enclosure were equipped with climbing structures and enrichment devices (food puzzles, boxes, etc.). Macaques were fed daily with assorted fruits and vegetables, nuts, seeds and commercial monkey pellets. Water was available ad libitum.

### Procedure

Data were collected during four mutually exclusive conditions (Table. 1). Although there was a predetermined sampling time for each condition on any given observation day (Table. 1) the start time of each sampling condition was evenly distributed to account for any circadian variation and to allow appropriate comparison between the conditions. Continuous focal animal sampling (10 minute focals) was used to collect data on self-directed and social behaviours, scan sampling (2 minute intervals) was used to record resting and moving behaviours [17] (Table. 2). Due to the layout of the enclosure and the macaques favouring the indoor sections, focal subjects rarely moved out-of-sight from the observer, but during these rare events the current focal was terminated and repeated at the next available opportunity. Group scan samples (5 minute intervals) were used to record the proximity of all visible individuals to each other in the group [17]. Behavioural observations were conducted by JW, LP, and CB, and proximity scans conducted by JW and LP. Interobserver reliability analysis using Cohan’s kappa [18] was used to assess for conformity to the coding system between each of the observers. Significant agreement was found between JW and LP (Behavioural coding, *n* = 20, k = 0.86, *P*<0.001; Proximity scan coding, *n* = 20, k = 0.90, *P*<0.001) and JW and CB (Behavioural coding, n = 30, k = 0.77, *P*<0.001).

**Table 1:** Description of sampling conditions and contexts, including total observational time.

Condition	Hrs of observation	Definition
Non-testing	30.17	Observations taken on days where no cognitive testing takes place. Baseline condition.
Pre-testing	25.50	Observations taken within 2 hours prior to a scheduled cognitive testing session.
Post-testing	30.83	Observations taken within 2 hours after a scheduled cognitive testing session.
During-testing	10.00	Observations taken of group mates during the testing of another individual. Samples taken opportunistically. Length of samples varied as voluntary testing sessions varied with subject motivation.

**Table 2:** List of behaviours monitored during all conditions.

Behaviour	Definition
Scratching (FS)	Repetitive raking of the skin or hair using fingers or feet.
Self-grooming (FS)	The cleaning of the skin or hair of itself. Hair is brushed as parted using the hands, hair or particles picked up with the hand or mouth.
Social grooming (FS)	The cleaning of the skin or hair of a partner. Hair is brushed and parted using the hands, particles picked up with the hand or mouth. Can be performed passively or mutually.
Lipsmacking (FS)	Rapid lower jaw movement with pursed lips. Often with scalp retraction and flattened ears. Used during affiliative interaction.
Aggression (FS)	Includes engaging in conflict, or displaying open mouth threats.
Resting (SS)	Individual remains stationary in a rested posture, with or without eyes closed.
Moving (SS)	Individual engages in any activity or behaviour. A non-resting individual.

FS = recorded during focal samples, SS = recorded during scan samples.

### Behavioural Observations

The average time of focal observation for each individual was 19.29 hrs±0.50 (mean±SD). Rates of behaviours were calculated for each individual from focal samples (behaviour count divided by the total time observed). We chose sociopositive behaviours (social grooming, lipsmacking), socionegative behaviours (aggression) and behaviours linked to stress of varying intensities (high intensity scratching and low intensity self-grooming) for comparisons. Although there may be taxonomic differences in how stress affects self-directed behaviours in this understudied species, current available data suggests their reactions to stress in this way are comparable to the rest of *Macaca* [19]. Rates of resting were calculated for each individual from scan samples (scans observed resting divided by total scans collected). The Wilcoxon-signed Ranks test was used to compare rates of behaviours between each of the sampling conditions, avoiding pseudoreplication by conducting within-subject analyses. This test provides power advantages over other non-parametric tests when working with small sample sizes, and also due to a small sample size probability values are accompanied with a standardized effect size (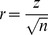) [20]. Significance level was set at a 0.05 and SPSS 20 was used for analysis.

### Social Network Analysis

A proximity measure for each dyad was calculated from scan sample data. This value was the frequency in which individuals A+B were recorded within 1 meter of each other divided by the total number of scans either A or B were visible. Interaction measures for each dyad were calculated from focal sample data. This value was the frequency in which individuals A+B interacted divided by the total time A and B were observed as focals. Any individuals who were currently participating in cognitive tasks were not included during proximity scans and their absence was recorded during focals. This was then taken into account to produce measures which were relative to the amount of time it was possible for two individuals to interact. We investigated affiliative interactions (grooming and lipsmacking) and agonistic interactions (conflict and threats). This provided square matrices of association and interaction data.

Statistical properties of the social network were calculated using UCINET 6.0 [21]. Centrality of each group member was calculated from association and interaction matrices using degree centrality measures, a value derived from the strength of overall connections with other individuals [7]. Wherever appropriate, individual centralities were assessed for correlation between conditions using Spearman’s Rank correlation coefficient to investigate changes in ‘popularity’ throughout the group. Kendall row-wise matrix correlations were used to calculate correlation between the proximity and association matrices; Matrix tester was used for analysis with 5000 permutations [22], [23]. This analysis reused data, and therefore the statistical significance level was adjusted using the Bonferroni correction equation to reduce the likelihood of type I errors [24].

Uncorrelated matrices were further investigated using sociograms to visualise the changes in the social network. Multidimensional Scaling analysis was used to determine the position of each individual on the sociograms [25], values of centrality were used to determine the size of each node, and rates of association or interaction were used to determine the thickness of the edges between dyads. Caution over interpretations must be taken due to the small sample size. The graphical package used to construct sociograms was NodeXL [26].

## Results

### Effect of Cognitive Testing on the Behaviour

#### Non-testing vs. testing

Data were compiled from the three testing conditions (pre-, during- and post-testing) to form an overall testing day condition; this was in order to compare typical non-testing days to typical testing days (Table. 3). There was significantly more self-grooming behaviours on testing days (Wilcoxon Signed-Rank test, *Z = *−2.02, *P = *0.043, *r = *−0.90). No other changes in self-directed or social behaviours were observed when comparing these two conditions: Scratching (*Z = *−0.41, *P = *0.686, *r = *0.18), social grooming (*Z = *−0.674, *P = *0.500, *r = *0.30), lipsmacking (*Z = *−0.41, *P = *0.686, *r = *0.18) and aggression (*Z = *−1.483, *P = *0.138, *r = *0.66). Resting was not significantly different between these conditions (*Z = *−1.753, *P = *0.08, *r = *0.79).

#### During-testing vs. post-testing

Different conditions within a testing day were also compared to assess how rates of behaviour during a testing session compared to another period of the day (Table 3.). There were no changes in self-directed behaviours between testing and post-testing: scratching (*Z = *−0.67, *P = *0.500, *r = *0.30) and self-grooming (*Z = *−0.94, *P = *0.345, *r = *0.42). Rates of social grooming were also unchanged (*Z = *−1.21, *P = *0.225, *r = *0.54). However, we found rates of lipsmacking to be significantly higher in testing periods (*Z = *−2.02, *P = *0.043, *r = *0.90) and aggressive contact was significantly lower (*Z = *−2.02, *P = *0.43, *r = *0.90) There was no significant different in resting behaviours between these conditions (*Z = *−1.75, *P = *0.080, *r = *0.78).

**Table 3:** Rates of group behaviours per hour.

	Non-Testing vs. Testing		During-testing vs. Post-testing	
*Behaviour*	*Non-Testing*	*Testing*	*During-Testing*	*Post-Testing*
Scratching	18.66±4.68	19.92±5.10	16.44±7.62	18.82±5.52
Self-Grooming	5.58±1.68	7.26±1.62	7.38±2.58	5.94±2.04
Social Grooming	6.60±2.64	6.60±2.64	7.44±3.06	6.00±1.56
Lipsmacking	1.86±0.48	1.98±0.48	3.72±2.28	0.84±0.78
Aggression	0.90±0.60	1.32±0.72	0.54±0.48	1.46±0.94

Values are Mean±SD, N* = *5.

#### Social network analysis with proximity measures

**Figure 1:**
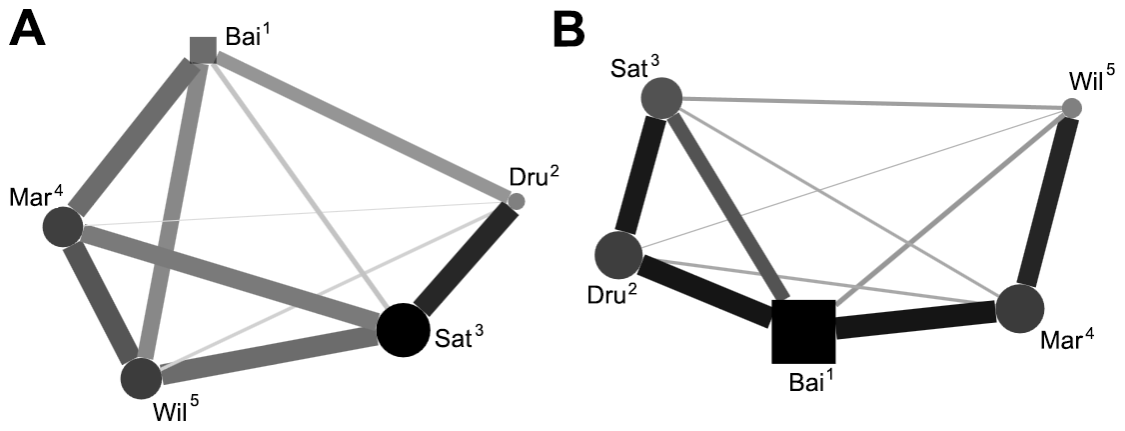
Between day comparison of spatial association (≤1 m). Sociograms comparing A) non-testing conditions and B) testing conditions. Nodes represent individuals in the group (n = 5), node shape indicates sex (square, male), node size and opacity represents centrality, edges represent dyadic association (rates of and edge thickness represents rates of association. Positions of nodes determined through MDS analysis. Numbers represent position in the hierarchy.

**Figure 2:**
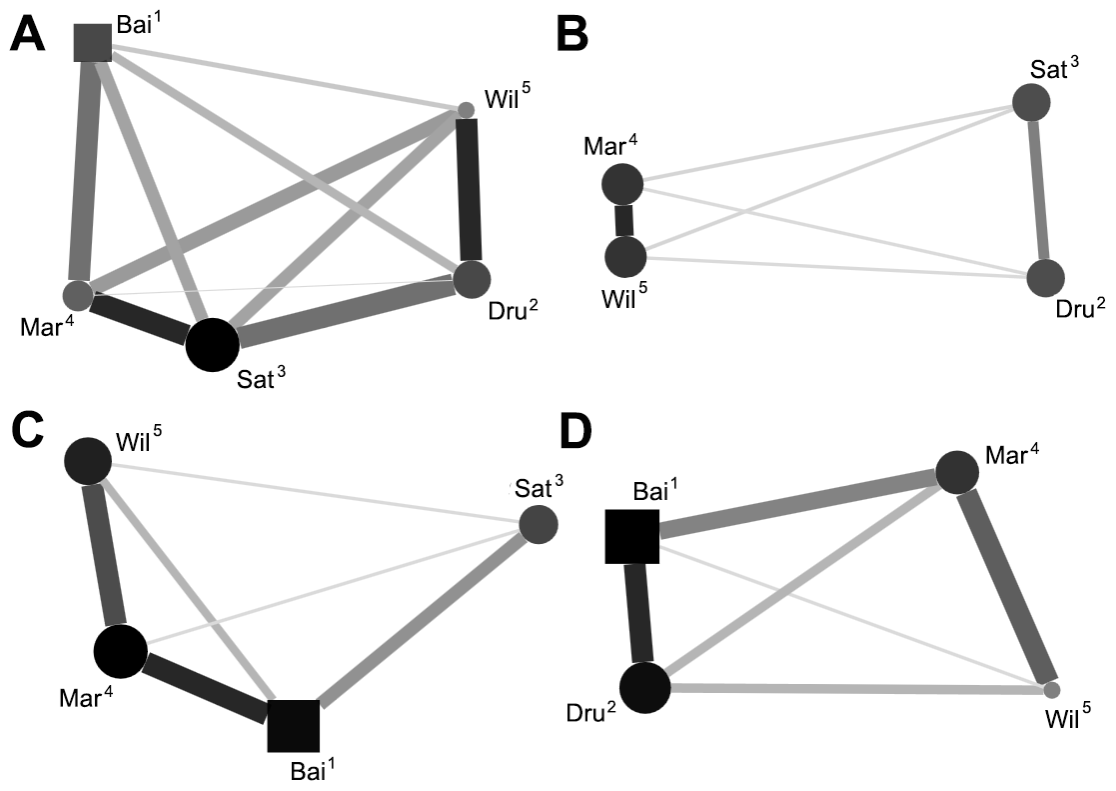
Within day comparisons of spatial association. Sociograms comparing A) post-testing condition, B) during the testing of individual Bai, C) during the testing of individual Dru and D) during the testing of individual Sat. See Fig. 1 for details. The individual who split from the group to participate in cognitive testing is not included in the social networks B, C, and D.

Average association (frequency in which two individuals were observed within 1 m proximity to each other) matrices were assessed for correlation between each of the conditions. Due to reuse of data in these analyses, significance level was adjusted to 0.025 (Bonferroni correction; 0.05/2 = 0.025). Positive correlation of the matrices were not found when comparing testing and non-testing conditions (Kendell row-rise matrix correlation, τKr* = *0.40, *P = *0.103). Associations were also not correlated between During-testing and post-testing conditions (τKr* = *0.47, *P = *0.045). We further investigated the uncorrelated conditions to assess for changes in degree centrality of subjects (n = 5). There was no significant correlation of centrality between testing and non-testing conditions (Spearmans, *RS = *0.60, *P = *0.285) and no correlation between during-testing and post-testing conditions (*RS = *0.05, *P = *0.935); this suggests that periods of testing are affecting the likelihood of certain individuals becoming involved in associations. Sociograms comparing social networks of the uncorrelated conditions were constructed to further interpret these changes (Fig. 1, Fig. 2).

#### Social network analysis with behavioural measures

**Figure 3:**
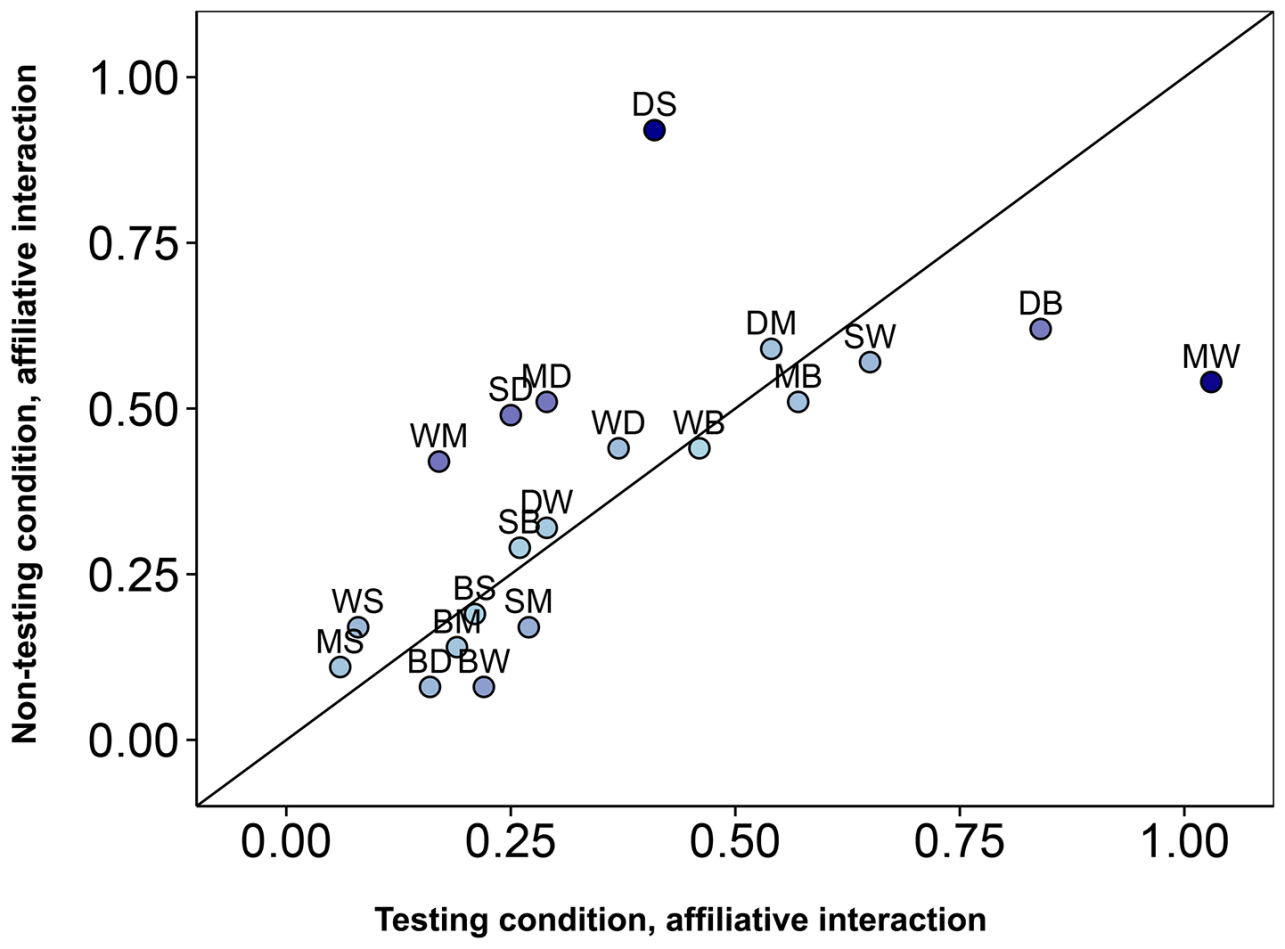
Correlated rates of affiliative interaction between days. This chart shows rates of affiliative interaction of all dyadic possibilities (n = 20). Each label AB is a dyad; were A is the actor and B is the recipient. Letters correspond to Bai, Dru, Sat, Mar and Wil. Dyads below the line show higher rates of affiliation during testing conditions and dyads above the line show higher rates of affiliation during non-testing conditions.

**Figure 4:**
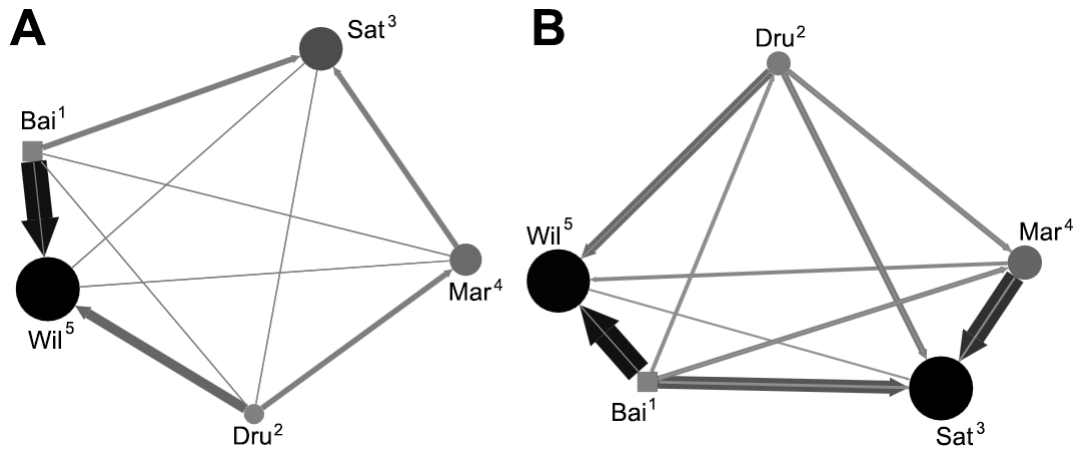
Within day comparison of aggressive interaction. Sociograms comparing A) non-testing conditions and B) testing conditions. See Fig. 1 for details. Instead here, node size and opacity represents in-degree centrality (received aggression) and edges and arrows represent the directed aggressive interaction and edge thickness represents rates of interaction.

Social network analysis was performed on two behavioural measures, affiliative interaction (rates of lipsmacking and grooming) and aggressive interaction (rates of conflict and threats). Matrices of average rates of behaviour were assessed for correlation between each of the conditions. Significant positive correlation of affiliative interactions was found between non-testing and testing conditions (Kendell row-rise matrix correlation, τKr* = *0.46, *P = *0.013) and during-testing and post-testing conditions (τKr* = *0.72, *P = *0.017). This suggests that overall social relationships in the group in terms of frequency of affiliative interaction were not influenced by testing. A scatter plot comparing all dyadic interactions between conditions was constructed to further investigate directions of affiliative interaction (Fig. 3). Significant positive correlation was found between aggression matrices when comparing testing and non-testing conditions (τKr* = *0.76, *P = *0.016). Due to the low frequencies of aggression in this group, multiple correlation analyses could not be done and data during the testing day conditions were only appropriate to use when combined. Sociograms were constructed to further interpret the direction of aggression throughout the group (Fig. 4).

## Discussion

Here we tested the impact of a cognitive research programme on a social group of zoo-housed primates (crested macaques). Increased group cohesion was observed during testing periods, in the form of decreased rates of aggression and increased rates of affiliation. Social network analyses also suggested a potential positive impact on welfare, as the observed changes in social dynamics in this study during testing days could be argued to be more comparable to populations of related species in the wild [27], [28]. There were also no increases in negative behaviours in the group as a result of cognitive testing. If welfare was being compromised, we would expect that the rates of normal social behaviours would be negatively affected during testing periods, so this is an encouraging result. For the most part, other rates of behaviour within the group remained comparable between non-testing and testing conditions which could also be interpreted as a good outcome in regards to welfare. If testing was impacting welfare negatively, scratching frequency could be expected to increase (this is due to the relationship between scratching and heightened stress [29]) but this was not found to be the case. Although it is difficult to conclude which specific part of the process could be producing the positive effects on the behaviour (the separation from the group, the tasks themselves, the additional contact with humans etc.) the overall process does not appear to have a negative impact on the social dynamics and hence the welfare of the group. Welfare may have been improved in this study, but an observed increase in self-grooming may cancel out the positive effects.

Self-grooming is also among the self-directed behaviours which have been described to be an indicator of stress [30], and here we found a slight increase in testing days. This could be interpreted as an increase in stress, although this would be more convincingly interpreted as such if this increase happened in synchrony to scratching behaviours. This is a pattern which occurs in many studies of stress related self-directed behaviour in primates [31], [32] and in fact other studies in chimpanzees have found self-grooming to be insensitive to increased anxiety [33]. Since scratching did not increase alongside self-grooming (it in fact decreased in frequency), an alternate interpretation of this change in behaviour may be necessary. It could be that self-grooming is a displacement of social grooming behaviour, due the increased separation of individuals from the group throughout a testing day. Although these conflicting findings are difficult to explain, all rates of self-directed behaviour remained at low frequencies during all conditions, despite the significant increase in self-grooming. It is also important to add that without the appropriate physiological data caution must be taken when making assumptions about the stress or anxiety of an individual, and such relationships between stress and self-directed behaviours are often complex and imperfect [32]. The absence of self-directed behaviours is therefore difficult to interpret as an individual free of stress. However, during a recent study comparable to this one but using saliva cortisol as measures of stress [34], similar positive effects on welfare as a result of voluntary testing were found.

Aureli and Yates [35] demonstrated that scratching behaviours can be alleviated through social grooming in crested macaques, which means grooming could potentially be used as a good indicator of changes in welfare in this species. There was no observed change in social grooming, lipsmacking behaviours, aggressive contact and resting behaviours between the days on which testing took place, and those on which it did not. This lack of change in social behaviour and resting provides evidence which suggests that the day to day welfare of our subjects was overall unaffected, either positively or negatively, by the cognitive testing.

When examining stress-related behaviours within a testing day, we found no differences between any of the conditions and both scratching and social grooming remained at comparable frequencies. However, an increase in affiliative lipsmacking and a decrease in aggression during testing sessions suggested a positive effect on the welfare of the group. The individuals who are actively involved in the cognitive tasks are the more dominant individuals, so it could be that lower ranking group mates are using this opportunity free from potentially higher ranking individuals to engage in affiliative interaction. Or alternatively, without the presence of dominant individuals to enforce order, increased affiliation may be necessary to prevent aggression. Increased aggression has been discussed as an indicator of poor welfare in animals [36] therefore decreased aggression could indicate improving welfare conditions. The reduction in conflict in this group is likely to have occurred simply as dominant individuals remove themselves for testing and therefore the most frequent aggressors are not currently integrated in the group.

Given this particular experimental design, although it is appropriate to interpret these changes in regard to possible changes in welfare, it is important to stress that these results are not limited to this single interpretation. Judge and de Waal [37] for example, present results comparable to this study however these were described as a response to situations of short-term crowding and were separate to issues of welfare.

Analysis of the social network found that cognitive testing sessions appeared to be changing the associations between individuals within the group with the emergence of more highly associated dyads on testing days. The splitting of individuals either singularly or in sub-groups due to conflicting motivations is now considered typical of most primate societies to varying degrees, including the macaques [38]. But the possibility for fission-fusion dynamics in captive primates is limited despite the demonstrated social benefits of group separation and reunion [39]. In a group of captive Tonkean macaques (*M. tonkeana*), De marco *et al.* [39] described behaviours of collective arousal – periods of heightened association as individuals who were separated experimentally were then reunited with their group. The differences in sociograms between the testing and non-testing days during this study could be interpreted as a product of these reunion behaviours. This could also help explain the increase in centrality by the two dominant individuals (Bai and Dru) who are both testing subjects and who each become more central in the group during testing days. The installation of cognitive testing equipment which is separated from the main enclosure area could allow conflicting motivations in the group to develop and promote basic forms of fission-fusion behaviour in captivity, which in turn could help replicate these social dynamics which are observed in wild macaques [27], [28]. This can be of great importance to welfare which can be defined in terms of how similarly captive and wild populations behave [40], and which is one of the main goals of animal husbandry.

The periodic absence of higher ranking individuals in the group during testing could be providing new social opportunities for the lowest ranking group mate, Wil. When observing the sociograms constructed from during testing data compared with the post-testing conditions (Fig. 2), Wil seems to become more integrated in terms of overall association during the testing of higher ranking group mates Bai and Dru (Fig. 2, B and C). This increase in association is not seen when the mid-ranking individual Sat (Fig. 2, D) leaves the group for testing. This could mean not only the reunion of the testing individuals back into the group can provide social benefits, but also the fission of higher ranking group mates away from the group could be providing benefits for the lower ranking individuals which typical captive conditions may not allow. Further studies of these reunion behaviours in other non-human primate species are needed. If these benefits are confirmed, better husbandry methods could be developed which facilitate subgroup separation and reunion and thus help increase the welfare of captive animals. Enclosure design, for example could take into account opportunities for individuals to separate themselves from the main group. Dominant individuals, however, may be less motivated to use such opportunities than subordinates (as subordinates are often located more peripherally) so providing enriching devices in isolated areas of enclosures may be useful to encourage this.

A possible increase in integration of the low ranking group mates can also be observed when a scatter plot of affiliative interactions is constructed between testing and non-testing days (Fig. 3). Lowest ranking group mates Wil and Mar become the pair who most frequently exchange in affiliative interaction. Changes in affiliative contact were not seen as typical throughout the entire group however, and overall rates of affiliation between dyads were correlated between the two conditions. So, despite the spatial differences observed between dyads (Fig. 1), cognitive testing may not highly disturb relationship quality.

Although rates of aggression did not significantly differ between testing and non-testing days, the direction of aggressive interactions changed. Although correlation of matrices suggested no significant redistribution of aggression, individual changes can be observed when further inspecting sociograms (Fig. 4). The cognitive testing equipment within the macaque study centre is for single use and is a resource for additional and unusual food items. It would be not be surprising if such a facility induced higher intragroup competition or monopolisation [41], [42]. Higher-ranking individuals tended to have a priority over the testing apparatus however this did not seem to increase the frequency of aggressive interactions. Instead, lower-ranking individuals left the testing unit when a higher ranking approached (i.e. supplantation, sensu [43]). Caution should be taken with this observation however as this may be species specific due to the crested macaques characteristically high social tolerance [44]. The sociograms (Fig. 4) show the majority of aggressive interactions are initiated by the dominant individual (Bai) and towards the lowest ranking individual (Wil) in both testing and non-testing conditions. Sociograms suggested a redirection of aggression was observed towards mid ranking testing subject (Sat) by a lower ranking individual (Mar). A tolerant dominance style is associated with this species [45], [46], and some small increase in aggression up the hierarchy may be fairly typical.

The benefits from the installation of cognitive testing equipment into zoo-housed primate enclosures are not limited to the researcher and their subjects, but there is also a good opportunity to engage with the public about science and enhance visitor experience and learning [47], [48]. Affiliations with zoos can also help to meet their educational and scientific aims [41] and thus should be encouraged. However local conditions will vary from zoo to zoo and consequently the influence of cognitive research on the welfare of subjects will vary widely too [6], it is therefore important that each circumstance is evaluated independently.

This study provides evidence to support the use of socially housed primates as a welfare positive alternative to laboratory housed primates for cognitive research. Although subjects were found to modify their behaviours and associations around cognitive testing sessions, many of these were short-term changes and may be typical of the social dynamics of the species in the wild (e.g. fission-fusion). It may be sensible to assume primates would avoid situations which are significantly detrimental to their psychological welfare and that voluntary participation by the subjects may itself be a positive sign. Participation of the entire social group is uncommon in group housed testing (this study, [11], [15]) but interestingly we have found that positive impacts were not limited to the participants and that testing may exert wider positive effects on the whole group. Therefore the observation of all individuals (through social network analysis) may be necessary to fully assess the welfare impact of cognitive testing in social groups. To be able to become more confident in the interpretations of these results however, future studies should include physiological data collected in parallel to observational behaviour which will allow much more accurate conclusions to be made about how cognitive testing affects welfare.
